# Mouse Invariant Monoclonal Antibody NKT14: A Novel Tool to Manipulate iNKT Cell Function *In Vivo*

**DOI:** 10.1371/journal.pone.0140729

**Published:** 2015-10-16

**Authors:** Felix Scheuplein, Deanna J. Lamont, Matthew E. Poynter, Jonathan E. Boyson, David Serreze, Lennart K. A. Lundblad, Robert Mashal, Robert Schaub

**Affiliations:** 1 NKT Therapeutics, Inc., Waltham, MA, United States of America; 2 The Jackson Laboratory, Bar Harbor, ME, United States of America; 3 The University of Vermont, Department of Medicine, Burlington, VT, United States of America; INEM, FRANCE

## Abstract

Invariant Natural Killer T (iNKT) cells are a T cell subset expressing an invariant T Cell Receptor (TCR) that recognizes glycolipid antigens rather than peptides. The cells have both innate-like rapid cytokine release, and adaptive-like thymic positive selection. iNKT cell activation has been implicated in the pathogenesis of allergic asthma and inflammatory diseases, while reduced iNKT cell activation promotes infectious disease, cancer and certain autoimmune diseases such as Type 1 diabetes (T1D). Therapeutic means to reduce or deplete iNKT cells could treat inflammatory diseases, while approaches to promote their activation may have potential in certain infectious diseases, cancer or autoimmunity. Thus, we developed invariant TCR-specific monoclonal antibodies to better understand the role of iNKT cells in disease. We report here the first monoclonal antibodies specific for the mouse invariant TCR that by modifying the Fc construct can specifically deplete or activate iNKT cells *in vivo* in otherwise fully immuno-competent animals. We have used both the depleting and activating version of the antibody in the NOD model of T1D. As demonstrated previously using genetically iNKT cell deficient NOD mice, and in studies of glycolipid antigen activated iNKT cells in standard NOD mice, we found that antibody mediated depletion or activation of iNKT cells respectively accelerated and retarded T1D onset. In BALB/c mice, ovalbumin (OVA) mediated airway hyper-reactivity (AHR) was abrogated with iNKT cell depletion prior to OVA sensitization, confirming studies in knockout mice. Depletion of iNKT cells after sensitization had no effect on AHR in the conducting airways but did reduce AHR in the lung periphery. This result raises caution in the interpretation of studies that use animals that are genetically iNKT cell deficient from birth. These activating and depleting antibodies provide a novel tool to assess the therapeutic potential of iNKT cell manipulation.

## Introduction

Invariant Natural Killer T (iNKT) cells have an invariant T Cell receptor (TCR), that in contrast to conventional T cells recognize glycolipid antigens that are presented on the MHC I like molecule CD1d. iNKT cells share surface markers and functional characteristics with both conventional T cells and natural killer (NK) cells [1,2]. iNKT cells represent a very small subset of the total T cell population in human and non-human peripheral blood. In humans, their abundancy ranges from less than 0.01% of all T cells to higher than 1.0%, with the majority of individuals clustering at the lower end of the range. In inbred mice iNKT cells are still a rare population but in the higher range of 0.5%-2% with very little within-strain animal to animal variation [3]. Despite their low frequency iNKT cells have potent immune-regulatory functions as they are constitutively expressing high levels of a wide variety pro-inflammatory as well as immune-regulatory cytokines and chemokines that are rapidly released upon iTCR engagement [2]. While iNKT cell activity has been implicated in the pathogenesis of asthma and inflammatory diseases such as sickle cell disease (SCD) [4,5,6,7,8,9,10], the pharmacologic activation of iNKT cells using glycolipid superagonist alpha-Galactosyl-ceramide (αGalCer) has shown them to be protective in the NOD mouse model of autoimmune Type 1 diabetes (T1D) [11,12]. The absence or functional defects in iNKT cell function has been demonstrated to lead to accelerated T1D onset in NOD mice [13,14], while iNKT cell activation has also been shown to boost anti-tumor immunity [15,16]. Therapeutic means to reduce or deplete iNKT cells could treat inflammatory diseases, while approaches to promote their activation may have therapeutic potential in certain infectious diseases, cancer or autoimmunity. NKT Therapeutics, Inc has developed an anti-human invariant TCR antibody that can deplete iNKT cells *in vivo* [17] that is currently in a Phase 1 trial in patients with SCD. To better understand the role of iNKT cells in preclinical models of disease we recently developed anti-mouse invariant TCR specific monoclonal antibodies. We report here that by modifying their Fc-portion these represent the first identified antibodies with a capacity to either deplete (NKT14) or activate (NKT14m) iNKT cells *in vivo* in otherwise fully immuno-competent mice. To validate the function of the antibodies tested their efficacy in the NOD model of T1D and in a mouse model of allergic airway inflammation and airway hyperresponsiveness (AHR) [5] a model of allergic asthma [7].

## Results

**Fig 1:**
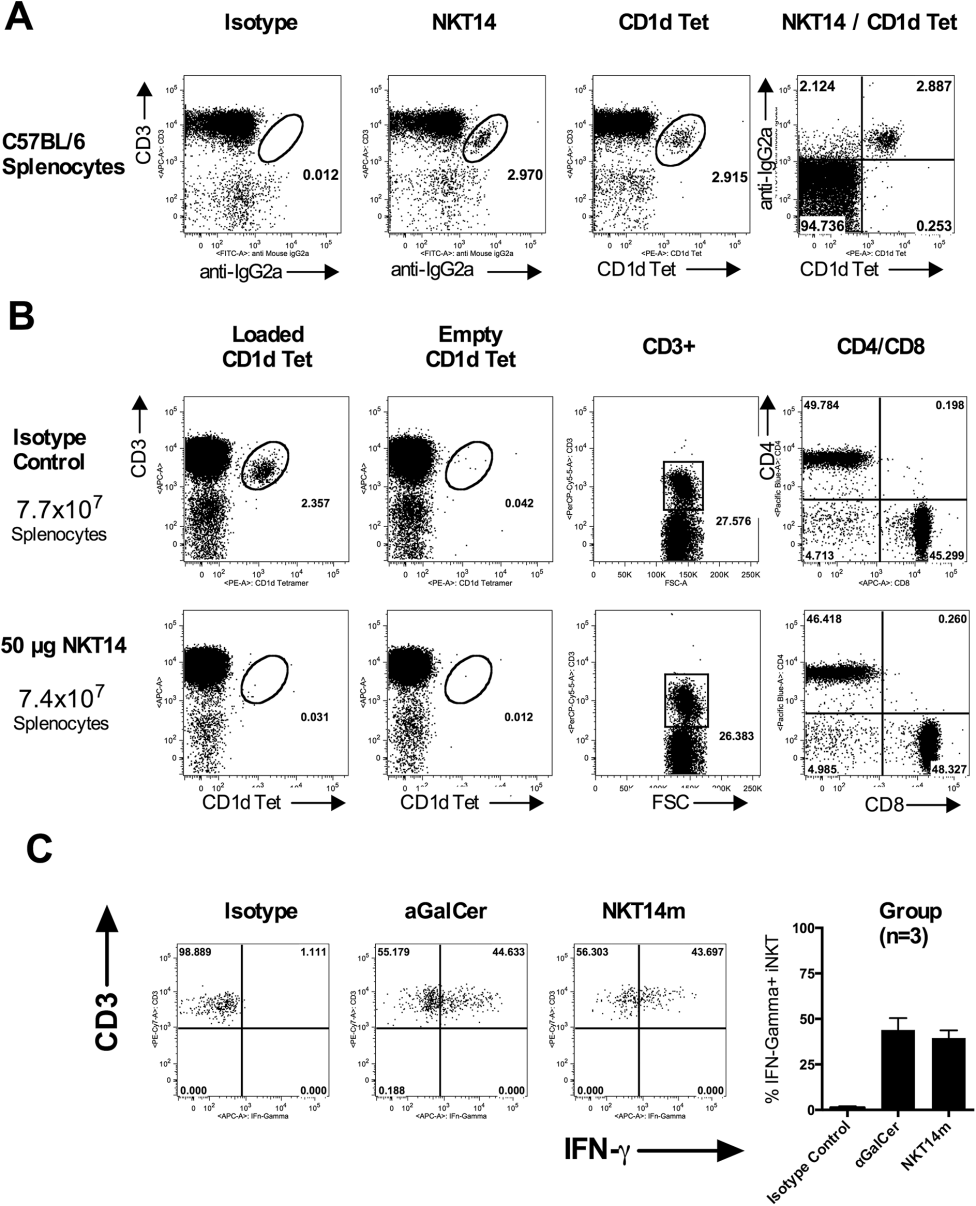
C57BL/6 splenocytes were stained with NKT14 or isotype control followed by a secondary anti mouse IgG2a monoclonal and αGalCer loaded CD1d Tetramer (A) The panel on the far right demonstrates co-binding of NKT14 and CD1d Tetramer. C57BL/6 mice (n = 3 per group) were injected i.p. with 50 μg NKT14 or isotype control. Mice were euthanized 24 hours after dosing and splenocytes were prepared, counted and stained for CD3, CD4, CD8 and aGalCer-loaded CD1d Tetramer to assess specificity of iNKT cell depletion. Data are representative of > 10 experiments (B). The CD4/CD8 ratio panel was gated on CD3+ cells. C57BL/6 (n = 3 per group) mice were injected with 2μg aGalCer, 50 μg NKT14m or isotype control. 2 hours post dosing mice were euthanized, splenocytes were prepared and stained for CD3 and αGalCer loaded CD1d tetramers to identify iNKT cells, washed, fixed, permeabilized and stained for intracellular IFN-Gamma. Representative FACS plots are shown as well as intracellular IFN-γ measurement across treatment group (C). Data are representative of >5 experiments.

Leveraging EUREKA Therapeutics’ proprietary human phage display library we identified a mouse invariant TCR specific antibody clone. Full length antibodies were made by cloning them in frame with either murine wild type IgG2a Fc to obtain a depleting antibody (NKT14) or an IgG2a with 4 point mutations to the Fc portion (L235E, E318A, K320A and K322A) to greatly reduce antibody dependent cellular cytotoxicity (ADCC) and complement-dependent cytotoxicity CDC function [18] for iNKT cell activation (NKT14m). NKT14 specifically binds to mouse iNKT cells identified by binding of αGalCer loaded CD1d Tetramers. Fig 1A shows co-binding of NKT14 to CD1d Tetramer positive iNKT cells in C57BL/6 splenocytes. There is no competition between CD1d Tetramer and NKT14, indicating different binding epitopes. We have tested multiple inbred mouse strains and confirmed this high specificity in the BALB/c, NOD, DBA, C3H, NZW, NZWxNZB, AKR and SJL strains (S1 Fig). NKT14 specifically depletes iNKT cells *in vivo* for approximately 3 weeks. 50 μg of NKT14 injected i.p. depleted iNKT cells without altering splenic lymphocyte count, T cell numbers or CD4/CD8 ratio (Fig 1B). Conversely, (and analogous to the αGalCer agonist) 50 μg of NKT14m injected i.v. rapidly activated iNKT cells, as indicated by measurable intracellular IFN-Gamma (Fig 1C), further upregulation of CD69 and upregulation of CD25 (S2 Fig).

**Fig 2:**
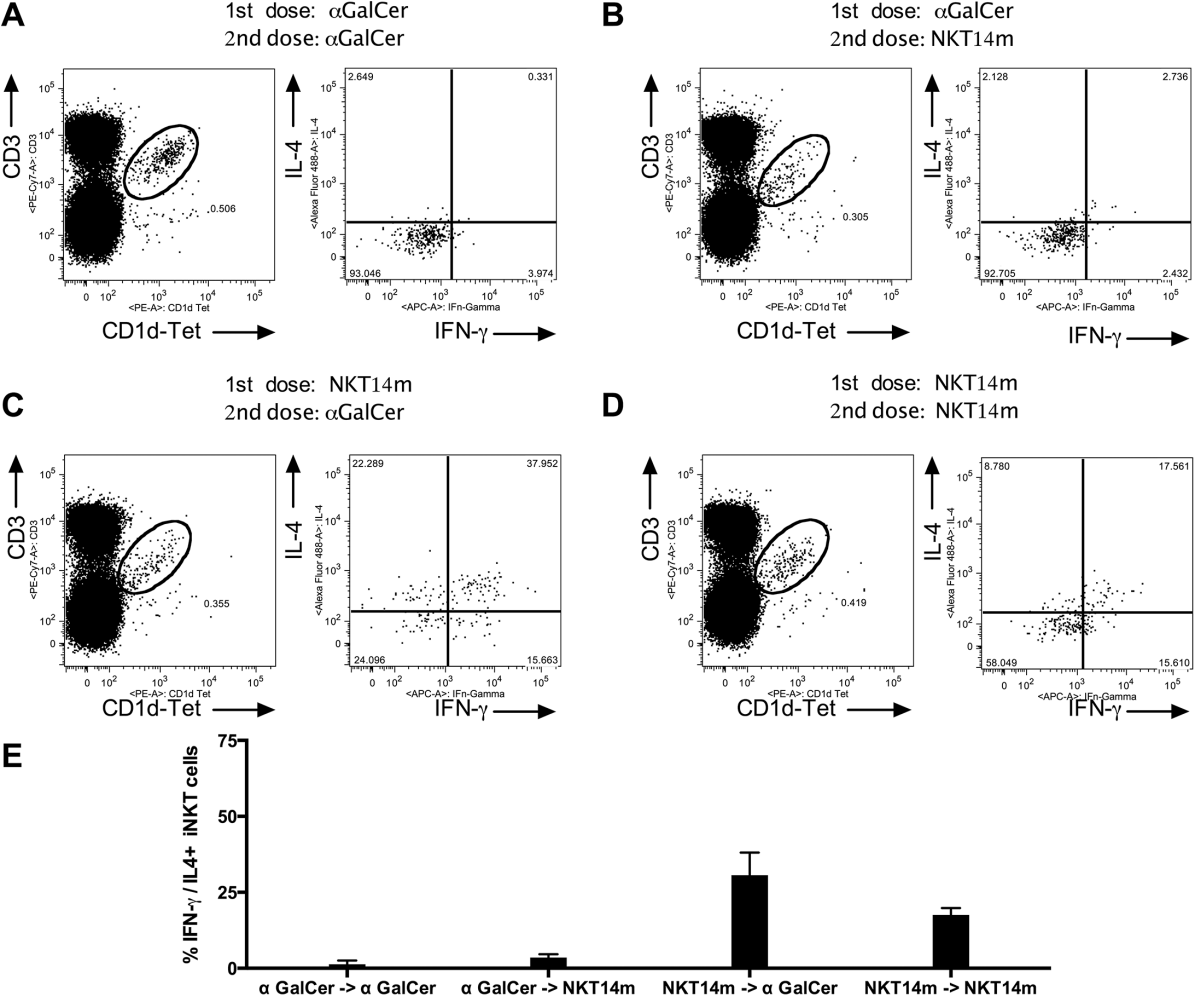
C57BL/6 mice (n = 3 per group) were injected i.v. with 50 μg NKT14m or i.p. with 2 μg αGalCer. 6 weeks after initial treatments mice were re-dosed with either NKT14m or αGalCer. Two hours after the second dose, mice were euthanized and splenocytes were analyzed by FACS for intracellular IFN-γ and IL-4. Mice were injected with αGalCer and re-challenged with αGalCer (A) or NKT14m (B) after 6 weeks. Mice were injected with NKT14m and then re-challenged with αGalCer (C) or NKT14m (D) after 6 weeks. % IFN-γ / IL-4+ iNKT cells across dosing groups (E).

We have used both the depleting and activating version of the antibody in the NOD model of T1D. To determine the best regimen for our activating antibody NKT14m, we did a repeat-dosing experiment. The iNKT cell agonist αGalCer has been demonstrated to induce long term iNKT cell anergy. To assess anergy response and duration with our mAb we treated C57BL/6 mice with either αGalCer or NKT14m and then again 6 weeks later either with the same or reciprocal initially administered agent. As described in the literature, iNKT cells that have been activated with αGalCer are completely anergic to αGalCer or NKT14m after redosing at 6 weeks (Fig 2 top panel). In contrast, iNKT cells of animals that had been dosed with NKT14m were re-activated by either αGalCer or NKT14m (Fig 2 bottom panel). Based on this observation, we decided to dose every 6 weeks in the NOD T1D incidence study.

**Fig 3:**
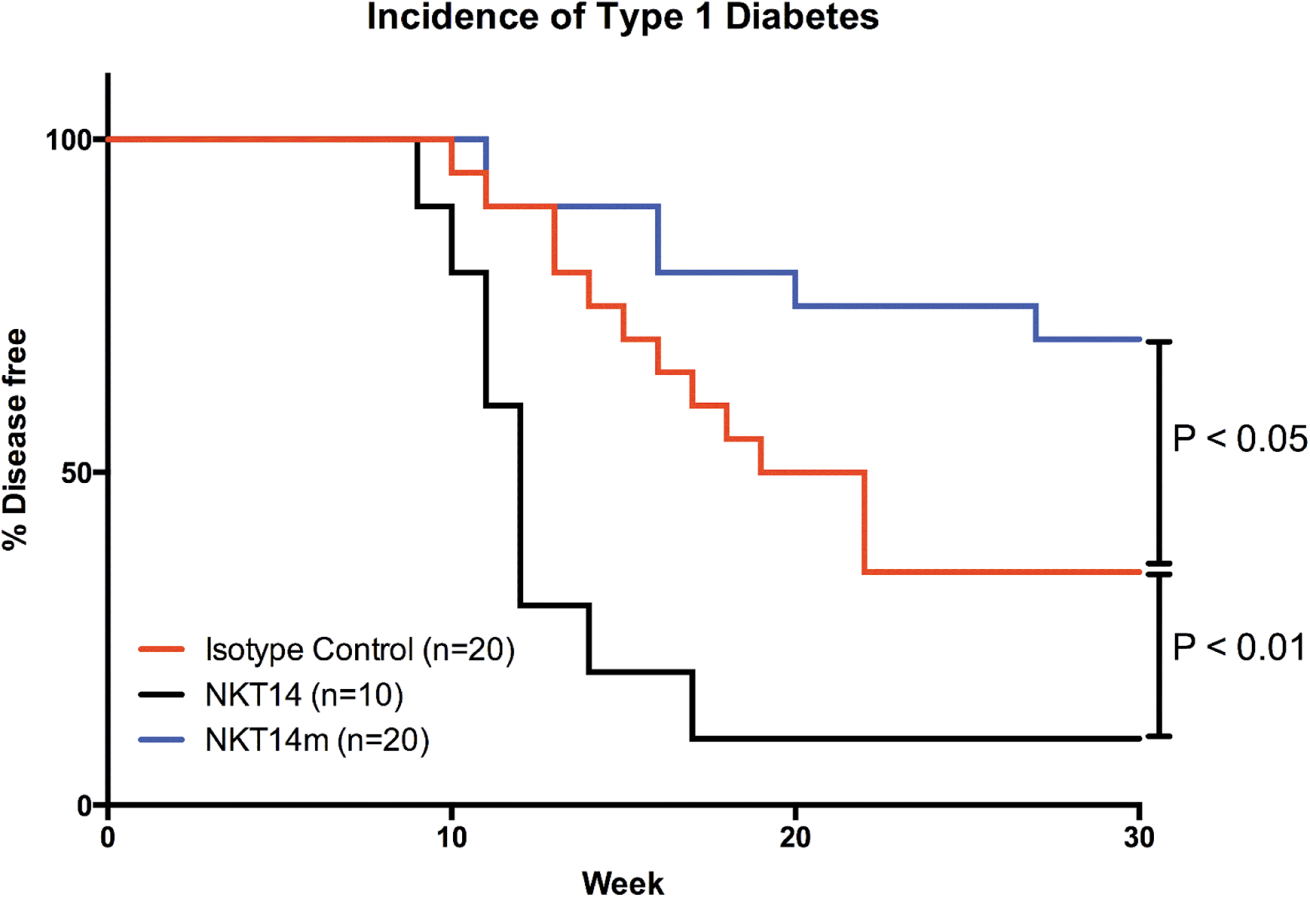
Starting at 4 week of age female NOD mice were injected i.p. every 6 weeks with the isotype control, NKT14, or NKT14m antibodies. Animals were monitored weekly for glycosuria. Positive animals (>300 mg/dL Glucose) were tested again the next day and euthanized if positive.

It has been reported that the absence or activation of iNKT cells respectively accelerates and retards T1D onset in NOD mice [11,13]. Starting at four weeks of age NOD mice were treated at 6 week intervals with isotype control (n = 20), NKT14 (n = 10), or NKT14m (n = 20) antibodies and monitored weekly for the inset of glycosuria. As predicted from the studies employing genetic ablation of CD1d expression, or the aGalCer iNKT agonist, NKT14 mediated depletion of iNKT cells led to significantly accelerated onset of T1D in NOD mice (P>0.01 Log-Rank (Mantel-Cox)), while their activation mediated by repeated administration of the NKT14m antibody partially protected and significantly delayed disease onset (P<0.05 Log Rank Mantel-Cox) (Fig 3).

**Fig 4:**
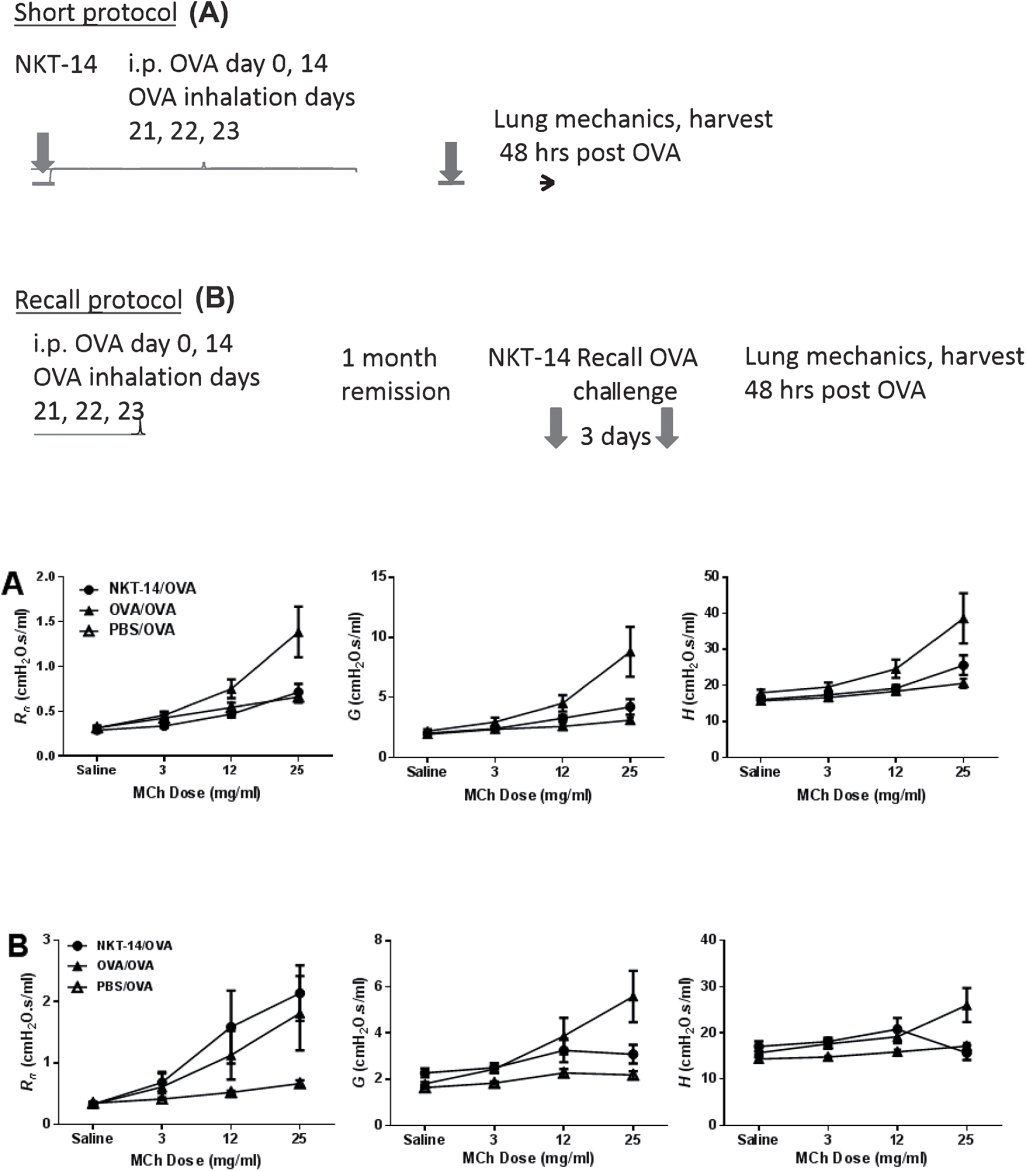
A) BALB/c female mice (8 per group) were treated with NKT14 (NKT-14/OVA) three days before being sensitized on days 0 and 14 with i.p. injections of OVA in ImjectAlum and then exposed to inhalational challenges with 1% OVA or control saline on days 21, 22, and 23. AHR was determined by methacholine challenge two days later. OVA/OVA are non-treated positive control mice and PBS/OVA mice were sham-sensitized negative controls. At the methacholine dose of 25 mg/ml NKT-14 treatment (NKT-14/OVA) significantly reduced the response in Rn compared with OVA/OVA (p<0.001); NKT-14 treatment also reduced the response in G (p<0.0001) and H, p<0.01) compared with positive control (OVA/OVA). B) Mice were sensitized on days 0 and 14 with i.p. injections of OVA in ImjectAlum and then exposed to inhalational challenges with 1% OVA or control saline on days 21, 22, and 23 and then again once after a 30 day recovery phase. AHR was determined by methacholine challenge two days later. iNKT cells were depleted with NKT14 three days before the final antigen challenge. At the methacholine dose of 25 mg/ml NKT-14 treatment (NKT-14/OVA) significantly increased the response in Rn compared with sham control (PBS/OVA) (p<0.01), whereas positive control (OVA/OVA) was significantly increased over PBS/OVA (p<0.05). However, the response in G was significantly reduced by NKT-14 compared with OVA/OVA (p<0.001). The response in H was also reduced by NKT-14 compared with OVA/OVA (p<0.001) Positive control group (OVA/OVA) was significantly increased over the sham control group (PBS/OVA) in G and H, p<0.0001 and p<0.001 respectively. Depletion of lung iNKT cells was confirmed by flow cytometry after euthanasia, but no differences in CD4+ or CD8+ cells were noted (S3 Fig). Analysis of Broncho alveolar fluid (BALF) found no differences in total cells across treatment groups. BALF eosinophils increased as both a percent and as absolute counts following sensitization. A small, but significant elevation of eosinophils was observed in NKT-14 treated mice compared to OVA/OVA mice (Data not shown).

To study the role of iNKT cells in a model of allergic airway inflammation and airway hyperreactivity (AHR) we treated BALB/c mice (n = 8/ group) with the iNKT cell depleting antibody NKT14 either before the sensitization phase, or just days before antigen re-challenge. When mice were treated before the sensitization phase, AHR to inhaled methacholine challenge was completely abrogated (Fig 4A). Conversely, untreated, but sensitized animals showed a significant AHR reaction. However, when iNKT cell depletion occurred in sensitized mice, briefly before the final antigen challenge, iNKT cell depletion completely failed to protect the mice from AHR in the conducting airways but significantly reduced AHR in the periphery of the airways as evidenced by reductions in *G* and *H* (Fig 4B).

## Material and Methods

### Mice and reagents

C57BL/6, NOD/LtDvs and BALB/c mice were purchased from the Jackson Laboratory. All *in vivo* experiments were approved by the IACUCs at the Jackson Laboratory, the University of Vermont and TGA Sciences (NKT Therapeutics Laboratory). Animals were euthanized by CO2 asphyxiation.

NKT14 and NKT14m were expressed in CHO cell lines and purified at Eureka Therapeutics. PBS57-loaded CD1d Tetramer was kindly provided by the NIH Tetramer facility. Fluorochrome labeled monoclonal antibodies targeting CD3 (17A2), CD4 (RM-4.4), CD8 (53–6.7), CD25 (PC61), CD69 (H1.2F3), IL-4 (11B11), IFN-γ (XMG1.2) and murine IgG2a FC (RMG2a.62) were purchased from Biolegend.

### Flow Cytometry

Single cell suspensions from spleens were generated by passing them in PBS/2% FBS buffer through a 70 μm Nylon filter (Miltenyi), processed with red cell lysis buffer (BD) and resuspended in FACS staining buffer (BD). 10^6^ cells were stained according to Biolegend recommendation, washed and analyzed on a LSR-Fortessa flow cytometer (BD). Data were analyzed using FLOWJO software (Treestar).

### T1D study

Beginning at 4 weeks of age, 20 NOD/LtDvs female mice were i.p injected with NKT14m or isotype control at 6 week intervals. A group of 10 four week old NOD/LtDvs female mice were i.p. injected with NKT14 in 6 week intervals.T1D development was assessed by weekly monitoring of urinary glucose levels with Ames Diastix (Bayer, Diagnostics Division), with disease onset defined by two consecutive values of >300 mg/dl.

### AHR Study

#### Sensitization with OVA

The mice were sensitized and treated according to either of two protocols; a Standard protocol and a Recall challenge protocol. Female BALB/c mice, 6 to 8 weeks old, were sensitized on Days 0 and 14 with intraperitoneal injections of 100μl ovalbumin (OVA) (20μg emulsified in 2.25 mg of alum, ImjectAlum (Pierce)) and then exposed to inhalational challenges with 1% OVA in saline or control saline as previously described (Days 21, 22, and 23) (19, 23). In the Standard protocol mice were tested for AHR on day 25. In the Recall protocol mice received a recall exposure to inhaled OVA one month after day 23 and were then tested for AHR two days later [19].

#### Assessment of airway hyperresponsiveness

48 hours after the last OVA inhalation exposure, the mice were anaesthetized with i.p. sodium pentobarbital (90mg/kg), the trachea cannulated and connected to a computer controlled small animal ventilator (flexiVent™, SCIREQ, Montreal, Canada), and ventilated at 200 breaths/minute. Next the mice were paralyzed with pancuronium bromide i.p. (0.8μg/kg). The depth of anesthesia was monitored with EKG throughout the experiment as previously described [20,21,22]. The animals were stabilized over about ten minutes of regular ventilation at a positive end-expiratory pressure (PEEP) of 3 cmH2O. A standard lung volume history was then established by delivering two total lung capacity maneuvers (TLC) to a pressure limit of 25 cmH2O and holding for three seconds. Following stabilization and standard lung volume history two baseline measurements of respiratory input impedance (Zrs) were obtained and then the mice were exposed to an inhalation of aerosolized PBS (vehicle control) for 10 s. Zrs was then measured every 10 s for 3 minutes. This complete sequence of maneuvers and measurements was then repeated for aerosol exposures to three incremental doses of methacholine (3.125, 12.5 and 25 mg/ml).

#### Respiratory mechanics

Zrs was determined using the forced oscillation technique. Briefly: Zrs over the frequency range 1–20.5 Hz was determined using a two second broadband perturbation in volume applied by the flexiVent™. Each determination of Zrs was fit with the constant phase model of impedance yielding the following parameters: Rn, the frequency independent Newtonian resistance reflecting that of the conducting airways, G characterizes tissue dampening, H characterizes tissue stiffness or elastance [23,24,25]. The peak response was identified as the average of the highest data point of each parameter, following each dose of methacholine, and the two data points immediately preceding and following the highest data point.

#### Statistics

Lung mechanics were tested for statistical significance using two-way ANOVA followed by Tukey’s multiple comparison test (Graph Pad Prism v6.04, GraphPad Software Inc. La Jolla, CA). Data is presented as mean ± SEM and p<0.05 was considered to be statistically significant.

## Discussion

We demonstrate here for the first time monoclonal antibodies specific for the mouse invariant TCR. NKT14 binds to the TCR of iNKT cells and can deplete them efficiently and specifically. This enables us to pharmacologically deplete iNKT cells at the time of choice in fully immunocompetent mice that were iNKT cell sufficient prior to treatment. We have modified the Fc portion of NKT14m to abolish ADCC and this antibody can activate iNKT cells without inducing long lasting anergy. We sought to test the antibodies in a mouse model setting where depletion or activation of iNKT cells had been previously reported to result in opposite disease outcomes. We chose the NOD model of T1D. Absence of iNKT cells leads to an accelerated onset of T1D in NOD mice [13,14], while their pharmacologic activation, or enhancement of their numbers inhibits disease onset [13]. Our monoclonal antibodies have the desired functions of depleting (NKT14) or activating (NKT14m) iNKT cells and that this translates into similar effects on T1D development in NOD mice as genetically ablating or pharmacologically activating this population. One of the disadvantages of genetically deficient iNKT cell mouse strains is limited specificity. CD1d deficient mice not only lack Type 1 invariant NKT cells, but also Type 2 diverse NKT cells and other CD1d restricted cells. Ja18 deficient mice recently have been shown to have a substantially reduced TCR repertoire [26]. Another possible disadvantage of CD1d deficient mice is that they lack iNKT cells from birth, so that it is difficult to elucidate the role of iNKT cells in either initiation or maintenance of a disease. iNKT cells are reported to be involved in development of AHR in mice [5] and have also been found in the lungs in the lungs of Asthma patients [7]. However, there have been no tools to test if iNKT cells are involved mostly in the sensitization leading to AHR or are actually causing the disease state. Our development of a specific iNKT cell depleting antibody allowed us to address this experimentally. AHR can manifest both in the conducting airways (increased Rn) as well as in the periphery of the lung (increased *G* and *H*). While the parameters are linked they can also change independently from each other [23]. In this study OVA mediated AHR of all parameters was abrogated by iNKT cell depletion prior to antigen sensitization. On the other hand, depletion of iNKT cells after initial sensitization reduced the AHR in the periphery of the airway system but no effect on AHR in the conducting airways. This observation suggests that iNKT cells could play a role for AHR in the airways and lungs with an established immunological memory. The different responses observed are also in keeping with our previously reported findings that AHR does manifest in geographically different regions of the lung depending on the sensitization/challenge time course and that AHR is an evolving phenotype [19]. Hence, it should perhaps not be surprising to find that the elimination of a specific part of the immune system, i.e. iNKT cells, displays different effects along the time course of allergy development. Incidentally there is evidence supporting that inflammation and AHR of the periphery of the lung are strong contributories to clinical asthma [27,28,29,30], which suggests that iNKT cells might be playing a role in the development of AHR in the distal parts of the lung.

The finding that AHR protection is absent when iNKT cells are depleted after sensitization, raises caution in the interpretation of studies that use animals that are iNKT cell deficient from birth. Although such studies can provide useful information regarding the role of health and disease, we feel that our antibodies provide an excellent tool to assess the therapeutic potential of iNKT cell manipulation.

## Supporting Information

S1 Fig Single cell splenocyte suspensions from NOD, DBA, C3H, NZW, NZBxNZB, AKR, SJ/L and BALB/c mice were prepared and stained with CD3 and NKT14 followed by anti-mouse IgG2a secondary or αGalCer loaded CD1d tetramers to identify iNKT cells. (TIF)

S2 Fig C57BL/6 (n = 3 per group) mice were injected i.v. with 50 μg NKT14m or isotype control. 2 hours post dosing mice were sacrificed, splenocytes prepared and stained for CD3 and αGalCer loaded CD1d tetramers to identify iNKT cells, washed, stained for cell surface CD25 and CD69, fixed, permeabilized and stained for intracellular IFN-γ. To demonstrate specific upregulation of CD69 and CD25, histograms were gated on B cells (black histograms) and iNKT cells (gray histograms). Bar graphs show upregulation across the group (S2A). C57BL/6 (n = 3 per group) were injected i.v. with 2μg αGalCer or 50 μg NKT14m. Serum cytokines were determined 2 hours and 24 hours post dosing. Serum cytokine response compared to aGalCer suggests that systemic release may be less robust and/or delayed (S2B).
(TIF)

S3 Fig BALBc mice treated with NKT14 had a significant reduction in lung iNKT cells compared to other experimental groups. However no changes in CD4+ or CD8+ lymphocytes was observed in any of the experimental groups.
(TIFF)
