# Supplementary figures and images for: Mouse Invariant Monoclonal Antibody NKT14: A Novel Tool to Manipulate iNKT Cell Function In Vivo
Source: PLoS One. 2015 Oct 16;10(10):e0140729. doi: 10.1371/journal.pone.0140729 (PMC4608835; doi:10.1371/journal.pone.0140729)

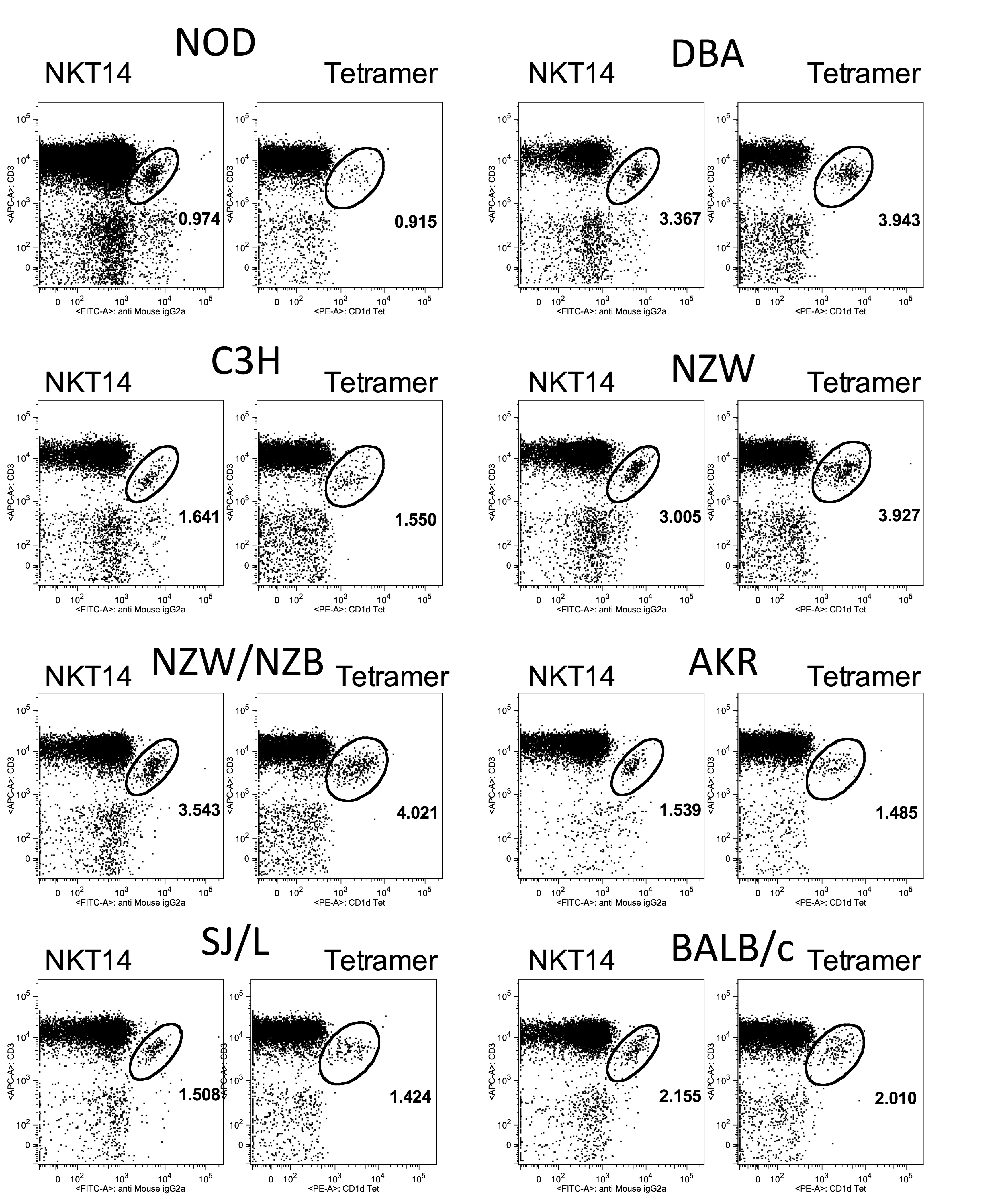

Supplement: S1 Fig — (TIF) [file pone.0140729.s001.tif]

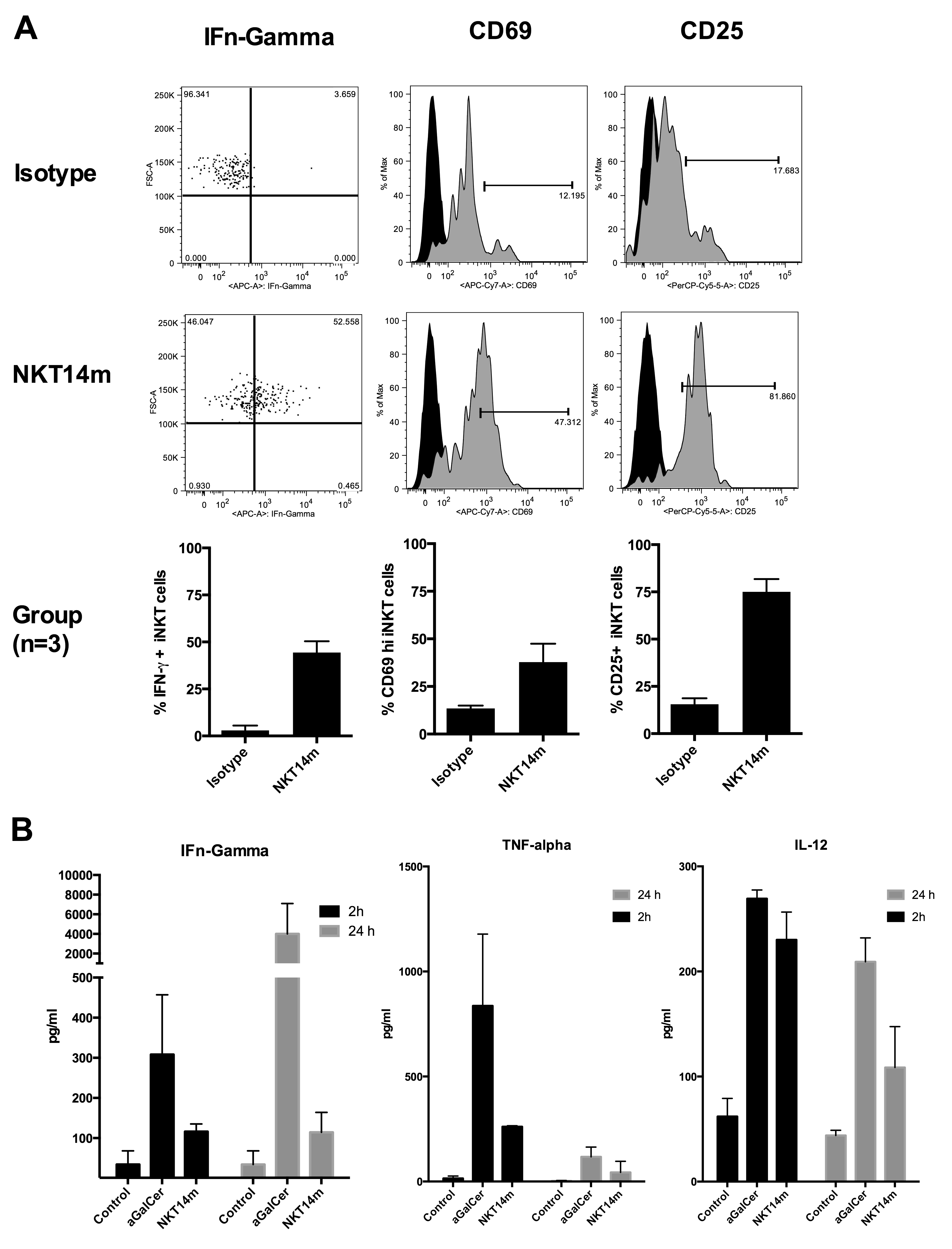

Supplement: S2 Fig — 2 hours post dosing mice were sacrificed, splenocytes prepared and stained for CD3 and αGalCer loaded CD1d tetramers to identify iNKT cells, washed, stained for cell surface CD25 and CD69, fixed, permeabilized and stained for intracellular IFN-γ. To demonstrate specific upregulation of CD69 and CD25, histograms were gated on B cells (black histograms) and iNKT cells (gray histograms). Bar graphs show upregulation across the group (S2A). C57BL/6 (n = 3 per group) were injected i.v. with 2μg αGalCer or 50 μg NKT14m. Serum cytokines were determined 2 hours and 24 hours post dosing. Serum cytokine response compared to aGalCer suggests that systemic release may be less robust and/or delayed (S2B). (TIF) [file pone.0140729.s002.tif]
